# Coevolution and Hierarchical Interactions of *Tomato mosaic virus* and the Resistance Gene *Tm-1*

**DOI:** 10.1371/journal.ppat.1002975

**Published:** 2012-10-18

**Authors:** Kazuhiro Ishibashi, Natsuki Mawatari, Shuhei Miyashita, Hirohisa Kishino, Tetsuo Meshi, Masayuki Ishikawa

**Affiliations:** 1 Division of Plant Sciences, National Institute of Agrobiological Sciences, Tsukuba, Japan; 2 Japan Science and Technology Agency (JST), Precursory Research for Embryonic Science and Technology (PRESTO), Kawaguchi, Japan; 3 Graduate School of Agricultural and Life Sciences, University of Tokyo, Yayoi, Bunkyo-ku, Tokyo, Japan; Universidad Politecnica de Madrid, Spain

## Abstract

During antagonistic coevolution between viruses and their hosts, viruses have a major advantage by evolving more rapidly. Nevertheless, viruses and their hosts coexist and have coevolved, although the processes remain largely unknown. We previously identified *Tm-1* that confers resistance to *Tomato mosaic virus* (ToMV), and revealed that it encodes a protein that binds ToMV replication proteins and inhibits RNA replication. *Tm-1* was introgressed from a wild tomato species *Solanum habrochaites* into the cultivated tomato species *Solanum lycopersicum*. In this study, we analyzed *Tm-1* alleles in *S. habrochaites*. Although most part of this gene was under purifying selection, a cluster of nonsynonymous substitutions in a small region important for inhibitory activity was identified, suggesting that the region is under positive selection. We then examined the resistance of *S. habrochaites* plants to ToMV. Approximately 60% of 149 individuals from 24 accessions were resistant to ToMV, while the others accumulated detectable levels of coat protein after inoculation. Unexpectedly, many *S. habrochaites* plants were observed in which even multiplication of the *Tm-1*-resistance-breaking ToMV mutant LT1 was inhibited. An amino acid change in the positively selected region of the Tm-1 protein was responsible for the inhibition of LT1 multiplication. This amino acid change allowed Tm-1 to bind LT1 replication proteins without losing the ability to bind replication proteins of wild-type ToMV. The antiviral spectra and biochemical properties suggest that *Tm-1* has evolved by changing the strengths of its inhibitory activity rather than diversifying the recognition spectra. In the LT1-resistant *S. habrochaites* plants inoculated with LT1, mutant viruses emerged whose multiplication was not inhibited by the *Tm-1* allele that confers resistance to LT1. However, the resistance-breaking mutants were less competitive than the parental strains in the absence of *Tm-1*. Based on these results, we discuss possible coevolutionary processes of ToMV and *Tm-1*.

## Author Summary

Viruses rapidly evolve and adapt to their host organisms, and the evolutionary processes can be reproduced in the laboratory (experimental evolution). In contrast, cellular organisms (that can be viral hosts) evolve much more slowly than viruses, but the fact that they have antiviral systems suggests that viruses and their hosts have coevolved. To explore the coevolutionary histories of viruses and their hosts, we focused on *Tm-1*, a *Solanum habrochaites* gene that confers resistance to *Tomato mosaic virus* (ToMV). Based on analyses of the *Tm-1* gene sequences in *S. habrochaites*, we demonstrated that a part of the gene has been under positive selection. Biochemical studies suggested that *Tm-1* has evolved to strengthen its inhibitory activity rather than to diversify recognition spectra. In addition, experimental evolution analyses suggested that overcoming the *Tm-1*-mediated resistance by ToMV is associated with fitness costs. Based on these results, we discuss how ToMV and the plant resistance gene have coevolved.

## Introduction

Because viral diseases often prevent plant reproduction, viruses affect the fitness of their host plants. To counter viruses, plants have developed defense systems such as gene-for-gene resistance and RNA silencing [1]–[5]. Viruses need to evade recognition by resistance genes and encode suppressors of RNA silencing for successful infection. This suggests that viruses and host plants have coevolved, although the processes remain largely unknown.

**Figure 1:**
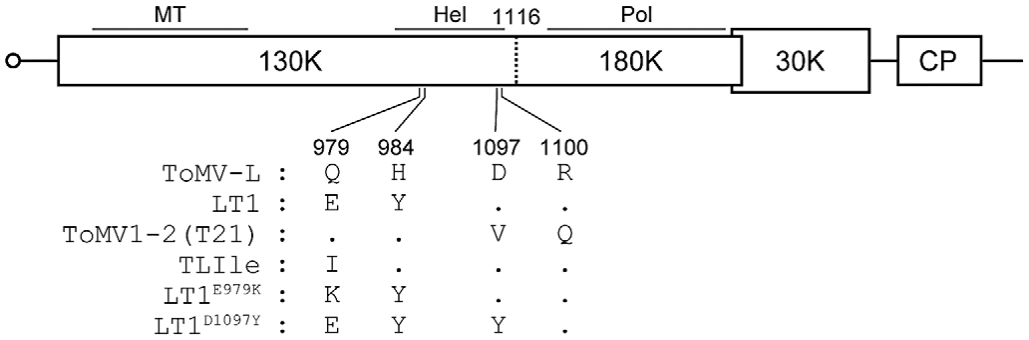
Schematic representation of ToMV mutant genomes with different sensitivities to *Tm-1* alleles. Positions of amino acid residue changes in *Tm-1*-resistance-breaking mutants are shown. Amino acid residues identical to ToMV-L are indicated by dots. LT1 and T21 are *Tm-1^GCR237^*-breaking mutants [20], [28] and TLIle is a *tm-1^GCR26^*-sensitive mutant [30], [50]. LT1^E979K^ and LT1^D1097Y^ were characterized in this study. MT: methyltransferase domain, Hel: helicase domain, Pol: RNA-dependent RNA polymerase domain, CP: coat protein.

*Tobacco mosaic virus*, *Tomato mosaic virus* (ToMV), *Tobacco mild green mosaic virus* (TMGMV), and *Pepper mild mottle virus* (PMMoV) are positive-strand RNA viruses belonging to the genus *Tobamovirus*. The tobamovirus genome encodes at least four proteins, namely, the 130K protein, the 180K protein (translational read-through product of the 130K protein), the 30K protein, and the coat protein (CP) (Figure 1). The 130K and 180K proteins are involved in RNA replication [6] and are collectively referred to here as replication proteins. The 130K protein is a multifunctional protein that interacts with many host proteins [7], as well as small RNA duplexes to function as a suppressor of RNA silencing [8]–[10]. The 30K protein is required for cell-to-cell movement [11], [12]. The CP is the only structural protein and required for systemic spread of the virus [13].

Several genes that confer resistance to tobamoviruses have been cloned, e.g., the *N* gene of tobacco [14], the *Tm-1* gene of tomato [15], the *Tm-2* gene alleles of tomato [16], [17], and the *L* gene alleles of pepper [18]. One of viral proteins is a determinant for resistance by each resistance gene; the 130K protein for *N* and *Tm-1* [19]–[21], the 30K protein for *Tm-2* [22], [23], and CP for *L* [24]–[26]. The frequency of emergence of resistance-breaking mutants varies from one resistance gene to another. For example, *N* and the *Tm-2^2^* allele of the *Tm-2* locus are durable, while mutant viruses easily overcome the resistance by *Tm-1*, the *Tm-2* allele of the *Tm-2* locus, and the *L* alleles. The resistance genes that are easily overcome may have evolved more rapidly, and thus they can be good targets of studying coevolutionary processes.

The *Tm-1* gene was introgressed from a wild tomato (*Solanum habrochaites* S. Knapp & D.M. Spooner) into tomato (*Solanum lycopersicum* L.) cultivars [27]. It encodes a protein that binds to ToMV replication proteins and inhibits RNA replication [15]. ToMV isolates that overcome the resistance conferred by *Tm-1* have mutations in the replication protein-coding region [20], [28] (Figure 1). A resistance-breaking mutant LT1 has replication proteins that do not bind the Tm-1 protein [15], suggesting that ToMV overcame *Tm-1* resistance by escaping the inhibitory interaction of its replication proteins with Tm-1. In the recently reported three-dimensional structure of the helicase domain of ToMV replication proteins, the residues involved in breaking the resistance are exposed to the surface of the molecule and locate in close spatial proximity [29], where Tm-1 likely binds. Translation product from a splicing variant of the *Tm-1* mRNA that lacks the second exon did not inhibit *in vitro* ToMV RNA replication, which indicates that a region in the Tm-1 protein encoded by the alternative exon is important for the inhibitory activity [15]. *Tm-1* homologs are widely conserved not only among plants, but also in fungi, bacteria, and archaea, suggesting that the Tm-1 protein has a primary function other than ToMV resistance and incidentally acquired the ability to bind ToMV replication proteins.

The ToMV-susceptible tomato cultivar GCR26 has a *Tm-1* allele, *tm-1^GCR26^*. The amino acid sequence of the tm-1^GCR26^ protein shows 97% identity with Tm-1^GCR237^, the product of the *Tm-1* gene from the ToMV-resistant tomato cultivar GCR237 [15]. The tm-1^GCR26^ protein does not bind the replication proteins or inhibit RNA replication of wild-type ToMV (L-strain) or LT1 [15]. However, tm-1^GCR26^ does bind the replication proteins and inhibit the multiplication of tobamoviruses that cannot infect tomato, namely, TMGMV, PMMoV, and the ToMV mutant TLIle in which the glutamine residue at position 979 of the replication proteins is replaced by an isoleucine residue [30] (Figure 1). TLIle, TMGMV, and PMMoV multiplication is also inhibited by Tm-1^GCR237^, indicating that tm-1^GCR26^ and Tm-1^GCR237^ have overlapping antiviral spectra [30].

Since most virus resistance genes are derived from wild relatives of the crops, studying the interactions between viruses and wild plants may elucidate the coevolutionary histories of viruses and plants. However, most molecular biological studies on plant resistance to viruses have been performed using crops or model plant species [31]. In this study, we analyzed ToMV resistance in *S. habrochaites* and show that a small part of the *Tm-1* gene has been under positive selection. We further identified a *Tm-1* allele that inhibits LT1 multiplication. On the other hand, evolution of microorganisms and their adaptation to hosts can be analyzed by experimental evolutionary methods in the laboratory [32]. In our experiments, ToMV mutants emerged that could overcome the LT1-resistant *Tm-1* allele, although the mutants were less competitive than the parental strains in the absence of *Tm-1*.

## Results

### Positive selection in the *Tm-1* gene of *S. habrochaites*

**Figure 2:**
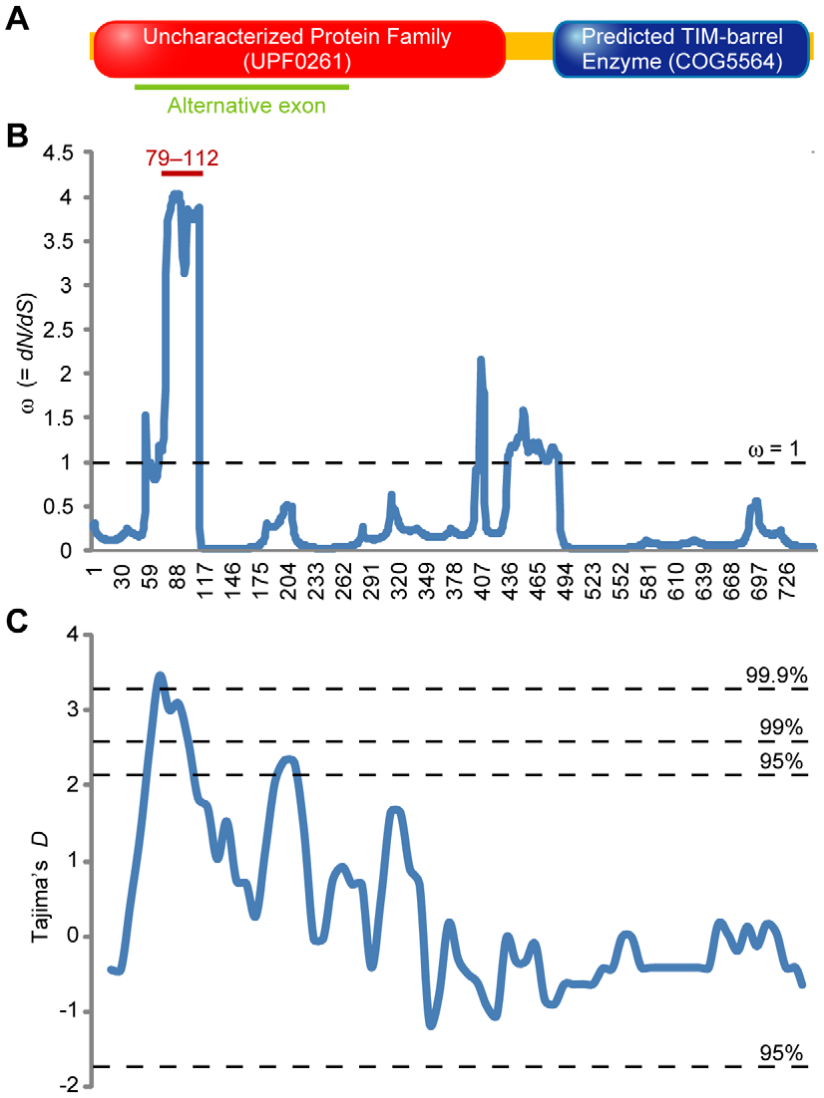
A small region of the *Tm-1* gene is under positive selection in *S. habrochaites*. (A) Predicted domain structure of the Tm-1 protein by the NCBI Conserved Domain Database. A region encoded by the alternative exon (46–263) is underlined. (B) Detection of natural selection in the *Tm-1* alleles from *S. habrochaites*. The ratio of nonsynonymous/synonymous substitutions (ω) in each codon was inferred by omegaMap [33]. ω>1, ω = 1, and ω<1 suggest positive selection, neutral evolution, and negative selection, respectively. The region where posterior probability of positive selection (ω>1) exceeds 95% is indicated (from 79^th^ to 112^th^ codon). (C) Sliding window analysis of Tajima's *D* of the *Tm-1* alleles from *S. habrochaites*. The confidence limits of *D* for neutral evolution [35] are shown as dashed lines.

To analyze the *Tm-1* gene of *S. habrochaites*, we obtained seeds of 24 *S. habrochaites* accessions from the Germplasm Resources Information Network (GRIN). All accessions were collected in South America (Peru, Ecuador, or Venezuela). From each accession, one plant was randomly chosen and the *Tm-1* cDNA was sequenced. In the obtained 48 sequences, a significant negative correlation was observed between linkage disequilibrium (*r^2^*) and distance between sites in the sequences (*r* = −0.2975, *p*<0.001), suggestive of intragenic recombination between alleles. Since this result indicated that the samples were not amenable for phylogenetic analyses, we used omegaMap [33] to analyze whether the evidence of natural selection is detected from the sequences in the presence of recombination. Remarkably, positive selection (ω = ratio of the rate of nonsynonymous/synonymous substitutions >1) was detected in a small region while most of the other parts of the gene were under purifying selection (ω<1) (Figure 2). An interdomain region (residues 432–483, predicted by NCBI Conserved Domain Database [34]) likely evolved neutrally (ω = 1) (Figure 2). The posterior probability of positive selection is >95% at residues 79–112. Consistently, Tajima's *D*, a test of neutral evolution [35], was significantly high (*p*<0.001) in the positively selected region based on a sliding window analysis (Figure 2C), also indicating that the region has not evolved neutrally. Importantly, the region is located in the alternative exon (encoding amino acids 46–263) of the *Tm-1* gene that is required for inhibitory activity (Figure 2) [15].

### ToMV resistance in *S. habrochaites*

**Figure 3:**
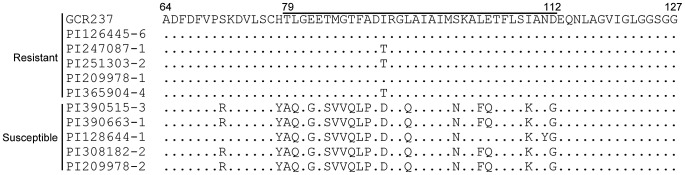
ToMV-L-resistant and -susceptible *S. habrochaites* have distinct amino acid sequences in the positively selected region of Tm-1. Deduced amino acid sequences of the Tm-1 protein of five ToMV-L-resistant and -susceptible *S. habrochaites* plants from the indicated accessions were aligned. The positively selected region (79–112) is indicated. Identical amino acid residues to those of Tm-1^GCR237^ are indicated by dots.

We next examined the ToMV resistance of 149 *S. habrochaites* plants from the 24 accessions by mechanically inoculating ToMV-L onto leaves. The accumulation of CP in the inoculated leaves was examined by SDS-PAGE, followed by Coomassie blue staining at 7 or 8 days postinoculation (dpi). Since *S. habrochaites* plants are self-incompatible, the accessions would not be genetically uniform. Indeed, in some accessions, both ToMV-resistant (CP undetectable) and -susceptible (CP detectable) plants were found (Table 1). Of the 149 plants tested, 94 did not accumulate detectable amounts of ToMV CP (Table 1). We then sequenced *Tm-1* cDNA of randomly chosen five plants that did not accumulate ToMV CP (i.e., ToMV-resistant plants) and five plants that accumulated ToMV CP at high levels (i.e., ToMV-susceptible plants). The amino acid sequences of the positively selected region were clearly divided into two classes, consistent with their ToMV-resistant or -susceptible phenotypes (Figure 3). In this region, each of the 48 Tm-1 amino acid sequences obtained above was similar to either one of the two groups (29 to the resistant type and 19 to the susceptible type; Figure S1). These results suggest that both types of alleles were maintained by balancing selection.

**Table 1:** Accumulation of ToMV-L CP in *S. habrochaites* accessions.

	Accumulation level of ToMV-L CP			
Accession numbers	+++^a^	+^b^	−c	Number of analyzed plants
PI126445	1	0	5	6
PI126446	1	2	3	6
PI127826	3	0	4	7
PI128644	7	1	0	8
PI209978	1	1	7	9
PI247087	0	0	6	6
PI251303	0	0	6	6
PI251304	0	0	5	5
PI308182	5	0	3	8
PI365903	0	1	4	5
PI365904	0	0	9	9
PI365905	0	0	5	5
PI365906	0	0	6	6
PI365907	2	1	2	5
PI379056	4	0	0	4
PI390515	6	0	0	6
PI390516	0	0	6	6
PI390517	1	0	5	6
PI390518	0	0	5	5
PI390658	2	0	5	7
PI390659	1	2	4	7
PI390661	3	2	1	6
PI390662	4	0	1	5
PI390663	4	0	2	6
Total	45	10	94	149

a high level accumulation,

b low level accumulation,

c not detectable.

In underlined accessions, more than 70% of plant individuals did not accumulate detectable amounts of ToMV-L CP.

In 10 out of the 149 plants, ToMV CP accumulated to low but detectable levels (Table 1). We sequenced the *Tm-1* cDNA of one such plant (PI390659) and found that the plant is heterozygous for the putative resistant and susceptible alleles. This result was consistent with a previous report showing that *Tm-1/tm-1* heterozygous tomato plants often permit delayed ToMV CP accumulation [36].

### LT1 resistance in *S. habrochaites*

To determine whether the *Tm-1* gene is responsible for the observed ToMV-L resistance, we selected 13 *S. habrochaites* accessions that contained relatively high proportions of ToMV-L-resistant plants (underlined in Table 1) and inoculated the *Tm-1^GCR237^*-resistance-breaking ToMV mutant LT1 onto 57 young seedlings from these accessions. For PI126445 and PI390658, CP accumulation was detected at high levels in three of four LT1-inoculated plants (Table 2). Note that *Tm-1^GCR237^* originated from PI126445. On the other hand, many plants in the other accessions showed only low or undetectable levels of CP accumulation in LT1-inoculated leaves (Table 2). This suggests that only a fraction of the ToMV-L-resistant *S. habrochaites* plants, including those in PI126445, are carriers of *Tm-1^GCR237^* or equivalent genes, and the rest have alternative or additional resistance factor(s) that prevent LT1 infection.

**Table 2:** Accumulation of LT1 CP in *S.habrochaites* accessions.

	Accumulation level of LT1 CP			
Accession numbers	+++^a^	+^b^	−c	Number of analyzed plants
PI126445	3	0	1	4
PI209978	2	1	2	5
PI247087	1	0	4	5
PI251303	1	0	3	4
PI251304	0	0	5	5
PI365903	1	2	1	4
PI365904	0	2	3	5
PI365905	0	2	3	5
PI365906	0	0	3	3
PI390516	2	0	3	5
PI390517	1	1	2	4
PI390518	0	1	3	4
PI390658	3	0	1	4
Total	14	9	34	57

a high level accumulation,

b low level accumulation,

c not detectable.

### Emergence of ToMV mutants that can multiply in LT1-resistant *S. habrochaites*

Although CP accumulation was undetectable at 8 dpi in approximately 60% of *S. habrochaites* plants inoculated with LT1 (Table 2), some of these plants showed disease symptoms at 15 dpi. In these plants, CP accumulation was observed. Since *Tm-1*-mediated resistance is easily overcome by mutations in the region coding for the helicase domain of ToMV replication proteins, the LT1 resistance in *S. habrochaites* may be due to a novel *Tm-1* allele and the accumulated viruses may have been resistance-breaking mutants. To test this hypothesis, we extracted RNA from six plants that showed delayed accumulation of CP (three from PI390516, one from PI390517, and two from PI390518), performed RT-PCR to amplify the helicase domain-coding region of ToMV, and sequenced. The sequences obtained from four plants had the same mutation at the key residue to overcome the *Tm-1*-mediated resistance (G3006 in LT1 to A). The 3006th nucleotide of the isolates from the other two plants remained as G, but we identified a mutation in another residue that was also important to break *Tm-1* (G3360 to T). Both G3006-to-A and G3360-to-T mutations cause amino acid substitutions (Glu979 in LT1 to Lys and Asp1097 to Tyr, respectively) (Figure 1). These findings strongly suggest that the observed LT1 resistance in *S. habrochaites* was conferred by an unidentified *Tm-1* allele.

### A single amino acid substitution in the Tm-1 protein confers the ability to inhibit LT1 multiplication

Based on the above results, we sequenced *Tm-1* cDNA isolated from three LT1-resistant plants from different accessions (PI251304, PI365904, and PI365906). Deduced amino acid sequences of the Tm-1 proteins of these plants showed differences from that of Tm-1^GCR237^ at several residues (Figure S2), among which three residues were common in the three LT1-resistant plants (Ile91, Leu408, and Asn452 of Tm-1^GCR237^ were changed to Thr, Phe, and Asp, respectively). Because Ile91 resides within the positively selected region, we speculated that the I91T substitution might be important for the LT1 resistance by Tm-1. We further sequenced Ile91-encompassing *Tm-1* cDNA fragments from an additional five LT1-resistant plants (PI247087, PI251303, PI390516, PI390517, and PI390518) and confirmed that they encode Thr at position 91.

**Figure 4:**
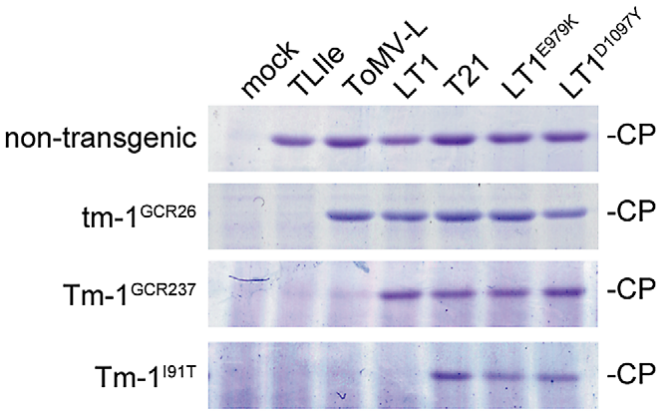
Tm-1^I91T^ inhibits the multiplication of LT1, but not LT1^E979K^ or LT1^D1097Y^. Protoplasts isolated from transgenic BY-2 cells expressing tm-1^GCR26^, Tm-1^GCR237^, or Tm-1^I91T^, or non-transgenic BY-2 cells were inoculated with TLIle, ToMV-L, LT1, T21, LT1^E979K^, or LT1^D1097Y^ by electroporation. At 20 hpi, protoplasts were harvested and coat protein (CP) accumulation was analyzed by SDS-PAGE and Coomassie blue staining.

To determine whether the Thr residue at position 91 is important for LT1 resistance, we prepared transgenic tobacco BY-2 cell lines, which constitutively expressed tm-1^GCR26^ protein, Tm-1^GCR237^ protein, or Tm-1 protein with the I91T substitution (Tm-1^I91T^). ToMV-LT1 cDNA was also mutagenized to encode an E979K (LT1^E979K^) or D1097Y (LT1^D1097Y^) substitution in the replication proteins to determine whether these mutations are responsible for overcoming the resistance by Tm-1^I91T^ (Figure 1). A ToMV-L mutant that has the same mutations as another *Tm-1*-resistance-breaking mutant (ToMV1-2) [28] was also constructed and named T21 (Figure 1). Protoplasts isolated from the transgenic BY-2 cells expressing tm-1^GCR26^, Tm-1^GCR237^, or Tm-1^I91T^, or non-transgenic BY-2 cells were inoculated with TLIle, ToMV-L, LT1, T21, LT1^E979K^, or LT1^D1097Y^ RNA by electroporation, or mock-inoculated, and CP accumulation was analyzed at 20 hours postinoculation (hpi). In non-transgenic BY-2 cells, the CP of these viruses accumulated to similar levels (Figure 4). In tm-1^GCR26^-expressing cells, multiplication of TLIle was inhibited (Figure 4). In Tm-1^GCR237^-expressing cells, multiplication of TLIle and ToMV-L was inhibited (Figure 4). In Tm-1^I91T^-expressing cells, multiplication of TLIle, ToMV-L, and LT1 was inhibited (Figure 4). Multiplication of T21, LT1^E979K^, and LT1^D1097Y^ was not inhibited by any of the Tm-1 variants (Figure 4). These results indicate that the I91T substitution in the Tm-1 protein confers the ability to inhibit the multiplication of LT1, while LT1^E979K^ and LT1^D1097Y^ emerged in LT1-resistant *S. habrochaites* plants by escaping from the *I91T*-type *Tm-1* alleles. Remarkably, sensitivity of ToMV mutants to Tm-1 variants was hierarchical; a virus that was unable to overcome tm-1^GCR26^ was also unable to overcome Tm-1^GCR237^ and Tm-1^I91T^, and viruses that were unable to overcome Tm-1^GCR237^ were also unable to overcome Tm-1^I91T^.

### Binding of ToMV mutant replication proteins with Tm-1 variants and inhibition of *in vitro* RNA replication

**Figure 5:**
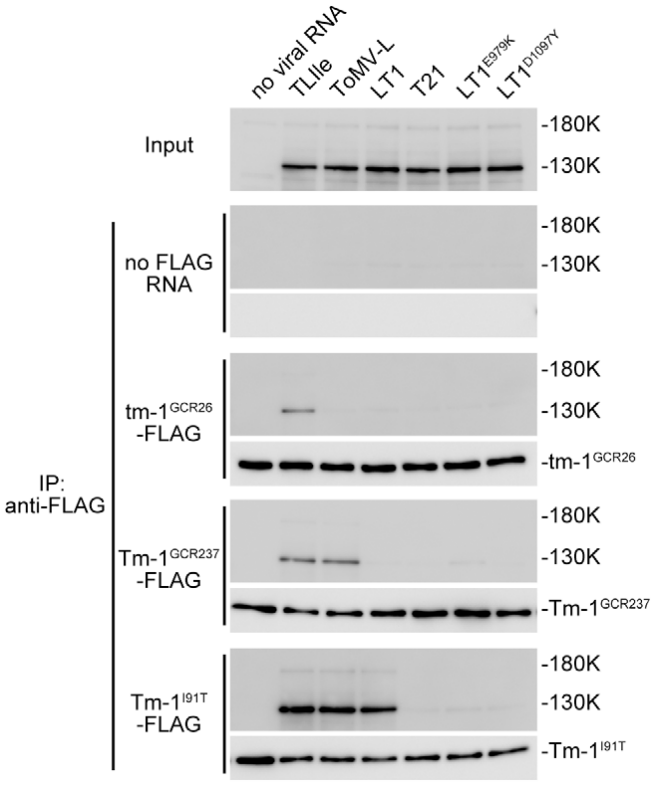
Tm-1^I91T^ binds LT1 replication proteins, but not LT1^E979K^ or LT1^D1097Y^. The genomic RNA of TLIle, ToMV-L, LT1, T21, LT1^E979K^, or LT1^D1097Y^ were translated in mdBYL; mixed with mdBYL in which tm-1^GCR26^-FLAG, Tm-1^GCR237^-FLAG, or Tm-1^I91T^-FLAG mRNA were translated; and immunoprecipitated using anti-FLAG antibody-conjugated agarose. Mock-translation was performed as controls and indicated as no viral RNA or no FLAG RNA. Protein samples before (Input) or after (IP: anti-FLAG) FLAG purification were analyzed by Western blotting using anti-130K protein or anti-FLAG antibodies. Positions of the replication proteins (130K and 180K proteins) and FLAG-tagged tm-1^GCR26^, Tm-1^GCR237^, or Tm-1^I91T^ proteins are indicated.

Tm-1 inhibits ToMV RNA replication by binding to the replication proteins [15]. Therefore, we examined the ability of Tm-1^I91T^ to bind LT1 replication proteins. FLAG-tagged tm-1^GCR26^, Tm-1^GCR237^, and Tm-1^I91T^ proteins were synthesized by *in vitro* translation using evacuolated tobacco BY-2 protoplast extracts from which membranes were removed by centrifugation (membrane-depleted BYL: mdBYL). The translation mixtures were mixed with mdBYL, in which TLIle, ToMV-L, LT1, T21, LT1^E979K^, or LT1^D1097Y^ RNA was translated or mock-translated, and immunoprecipitation using anti-FLAG antibody-conjugated agarose was performed. As expected, the LT1, TLIle, and ToMV-L replication proteins coprecipitated with Tm-1^I91T^-FLAG, while the LT1^E979K^ or LT1^D1097Y^ replication proteins did not (Figure 5). Also, the replication proteins of ToMV mutants whose multiplication was inhibited coprecipitated with the Tm-1 variants (Figure 5).

**Figure 6:**
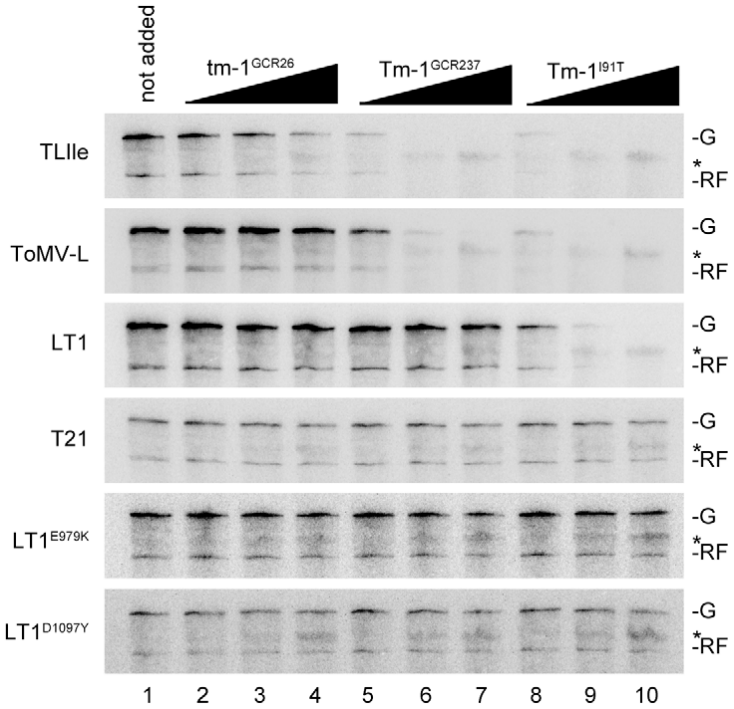
Inhibition of *in vitro* RNA replication of ToMV mutants by Tm-1 variants. The genomic RNA of TLIle, ToMV-L, LT1, T21, LT1^E979K^, or LT1^D1097Y^ and the mRNA for tm-1^GCR26^, Tm-1^GCR237^, or Tm-1^I91T^ proteins were translated in mdBYL. The translation mixtures of the Tm-1 variants were mixed with the viral RNA-translated mixtures, followed by RNA replication reaction as described in the Materials and Methods section. The amount of added Tm-1 mRNA were approximately 9 (lanes 2, 5, 8), 42 (lanes 3, 6, 9), or 126 (lanes 4, 7, 10) times as much as viral RNA on a molar basis. Mock-translated mixture was added as a control (lane 1). The positions of the genomic RNA (G) and the replicative form RNA (RF) are indicated. Asterisks represent the background signals.

We further analyzed the inhibitory effect of Tm-1 proteins on ToMV RNA replication using an *in vitro* ToMV RNA replication system [37], [38]. Briefly, ToMV RNA was translated in mdBYL. Tm-1 proteins were separately synthesized by *in vitro* translation with mdBYL, mixed with ToMV RNA-translated mdBYL, and incubated with BYL membranes and α-^32^P-labeled ribonucleoside triphosphates, followed by analysis of ^32^P-labeled RNA. Using this assay, we observed inhibition of RNA replication of the ToMV derivatives in a pattern consistent with the results of the protoplast experiment (Figure 6). Note that the inhibitory effect of tm-1^GCR26^ to TLIle RNA replication *in vitro* is weak [30]. Moreover, the *in vitro* experiment showed that (i) the inhibitory effect of Tm-1 variants is dose-dependent, (ii) TLIle is more sensitive to Tm-1^GCR237^ than ToMV-L (Figure 6, lanes 5–7), (iii) ToMV-L is more sensitive to Tm-1^I91T^ than LT1 (Figure 6, lanes 8–10), (iv) Tm-1^GCR237^ and Tm-1^I91T^ inhibit TLIle RNA replication more strongly than tm-1^GCR26^, and (v) Tm-1^I91T^ inhibits ToMV-L RNA replication more strongly than Tm-1^GCR237^ (Figure 6). These results suggest that I91T substitution in the Tm-1 protein strengthens its inhibitory activity enough to inhibit LT1 RNA replication, thus extending the antiviral spectrum.

### Fitness costs for ToMV to overcome resistance by *Tm-1* alleles

The observed hierarchical ToMV–Tm-1 interactions predict that LT1 or other resistance-breaking mutants should have emerged and dominated *Tm-1*-sensitive viruses in nature. However, many field isolates of ToMV from tomato are *Tm-1^GCR237^*-sensitive, although resistance by this gene was broken within a year of its introduction to commercial tomato cultivars in 1960s [39]. Thus, resistance-breaking mutants may have lower fitness than the wild-type in the absence of *Tm-1*. In fact, previous studies reported that a series of spontaneously isolated or nitrous acid-induced ToMV mutants capable of overcoming *Tm-1* (but not a field isolate) multiplied to lower levels and caused milder symptoms than wild-type virus in nonresistant *tm-1* tomato [40]. Also, a TMGMV mutant that can overcome resistance by *tm-1^GCR26^* (TMGMV-T894M,F970Y) had a compromised ability to suppress RNA silencing, an antiviral defense system of plants [41].

**Figure 7:**
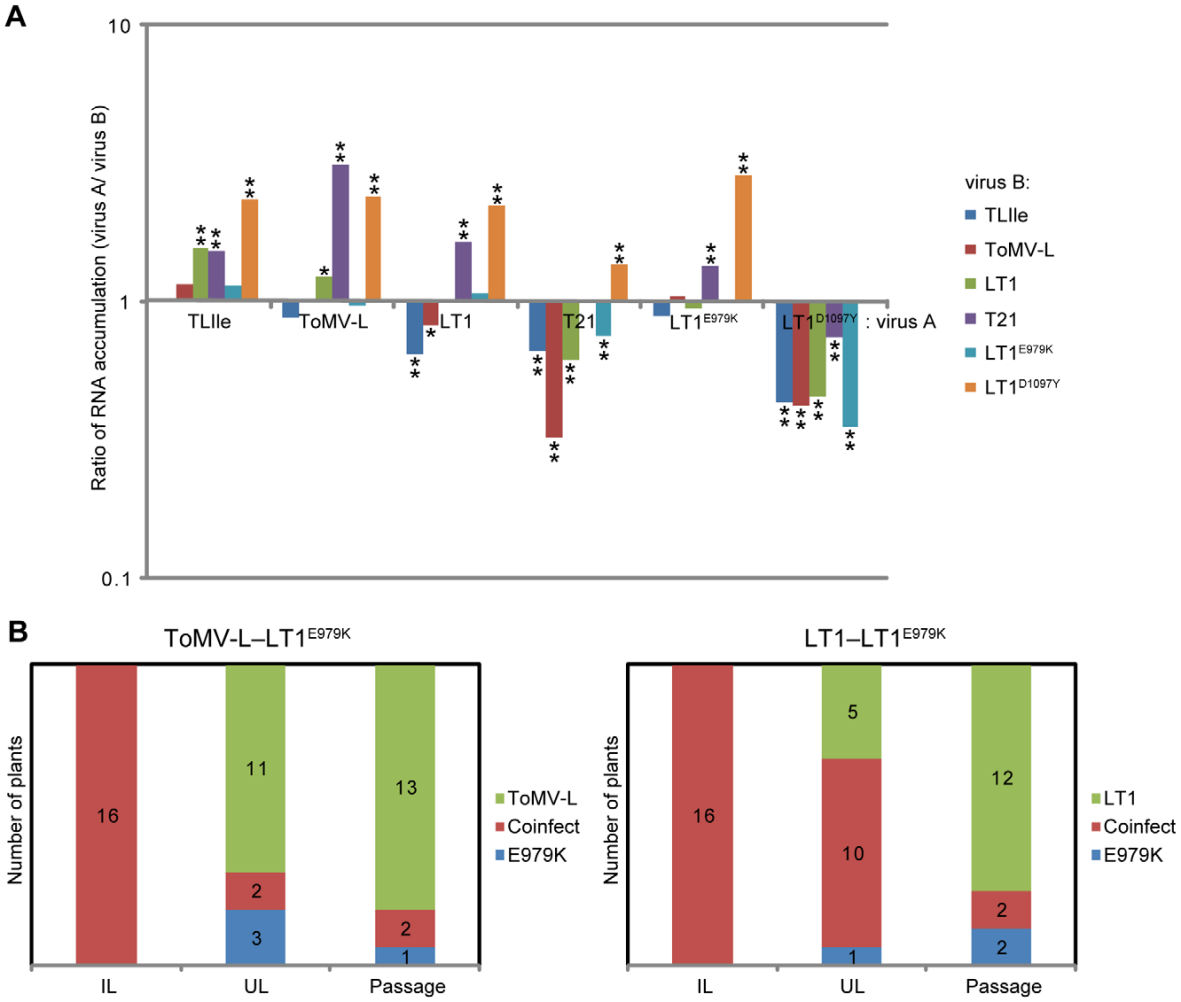
Fitness costs to the ToMV mutants in the absence of Tm-1. (A) Competition of two ToMV derivatives in BY-2 protoplasts. Protoplasts isolated from non-transgenic BY-2 cells were co-inoculated with two of the six ToMV derivatives used in this study. As a control, individual derivatives were separately inoculated and the protoplasts were cocultured. At 20 hpi, RNA was extracted, and amplified cDNA was sequenced using the GS-FLX titanium. Ratios of the viral count (virus A/virus B) normalized to the respective control experiment (individual infection) are shown. *: *p*<0.05, **: *p*<0.01 based on a chi-square test for the ratio of the two derivatives in the coinfection experiment against the ratio expected from the control experiment. A result of each competition is represented twice so that each virus A histogram shows the results of competition against all the other derivatives (virus B). (B) Competition of LT1^E979K^ with ToMV-L or LT1 in tomato plants. Mixtures of viral RNA were mechanically inoculated onto the leaves of 16 tomato (GCR26: *tm-1*) plants. RNA was extracted from the inoculated leaves (IL) and upper non-inoculated leaves (UL) at 10 and 42 dpi, respectively. At least four young leaflets of each co-inoculated plant at 46 dpi were homogenized; each homogenate was inoculated onto a healthy plant, and RNA was extracted from upper non-inoculated leaves at 42 dpi (Passage). RT-PCR-amplified cDNA fragments were directly sequenced and the numbers of plants accumulating either both or one of the co-inoculated derivatives are shown.

Although CP accumulation levels were not significantly different among the ToMV derivatives when they were individually inoculated into non-transgenic BY2 protoplasts (Figure 4), we examined the relative fitness between the ToMV derivatives by co-inoculation of BY2 protoplasts with a 1∶1 mixture of two ToMV derivative RNAs. As we had six ToMV derivatives, 15 combinations were tested. As a control, individual derivative RNAs were separately inoculated and the protoplasts were cocultured. At 20 hpi, RNA was extracted from the protoplasts and RT-PCR-amplified cDNA fragments of progeny viruses were sequenced by GS-FLX titanium (Roche, Basel, Switzerland). The ratio of the two strains in the progeny of the co-inoculation experiment was normalized to the control (individual) infection, and dominance by one of the two strains was examined using a chi-square test (Table S1). LT1^D1097Y^ was less competitive than the other five variants, as was T21 (excluding LT1^D1097Y^) (Figure 7A). Having amino acid substitutions at the same residue (D1097V for T21 and D1097Y for LT1^D1097Y^; Figure 1), the replication proteins of LT1^D1097Y^ and T21 would be disadvantageous with regard to multiplication within protoplasts, probably replicating the viral RNA. Similarly, LT1 RNA accumulation was lower than TLIle or ToMV-L RNA when co-inoculated (Figure 7A). Thus, LT1 is less competitive than ToMV-L and TLIle in the absence of *Tm-1*.

In contrast, LT1^E979K^ RNA accumulated to levels similar to those of TLIle, ToMV-L, or LT1 when co-inoculated (Figure 7A), suggesting that LT1^E979K^ is not at a disadvantage in protoplasts. Thus, we performed co-inoculation experiments of LT1^E979K^ with ToMV-L or LT1 to 16 tomato plants (cv. Craigella GCR26; *tm-1/tm-1*). RNAs were purified from the inoculated leaves at 10 dpi and from upper non-inoculated leaves at 42 dpi. RT-PCR-amplified viral cDNA fragments were sequenced to determine whether both of the inoculated strains accumulated or if one strain was eliminated. In the inoculated leaves, all plants accumulated both of the co-inoculated strains (Figure 7B). In upper non-inoculated leaves, only two (for ToMV-L–LT1^E979K^) and 10 (for LT1–LT1^E979K^) co-inoculated plants showed coinfection, while the remainder of the plants accumulated only one of the strains (11∶3 for ToMV-L∶LT1^E979K^, 5∶1 for LT1∶LT1^E979K^) (Figure 7B). Next, viruses in the young leaves of these 16 co-inoculated plants were reinoculated to uninfected plants. After the passage, LT1^E979K^ was eliminated in 13 or 12 of 16 inoculated plants in competition with ToMV-L or LT1, respectively (Figure 7B). From the results, we estimated relative fitness values for ToMV-L and LT1 against LT1^E979K^ as 4.31±0.05 and 2.43±0.07, respectively (for details see Text S1 and Figure S3). Thus, the replication proteins of LT1^E979K^ are likely to have a compromised function required for virus spread in plants. Taken together, the ability to overcome Tm-1^GCR237^ or Tm-1^I91T^ by ToMV is accompanied by pleiotropic fitness costs, i.e., impaired RNA replication in single cells or virus spread in plants.

## Discussion

Since viruses cannot multiply without a host cell, they evolve under selective pressure imposed by their hosts. In contrast, little evidence exists that wild plants coevolved with viruses [31]. A notable exception is the eukaryotic translation initiation factor (eIF) 4E gene in *Capsicum annuum* and other plants [42]–[44]. For potyviruses, a successful interaction between the viral protein VPg and eIF4E is required for virus multiplication, and disruption of this interaction results in resistance. Thus, mutations in the *eIF4E* gene that affect the interaction with VPg confer recessive resistance to the corresponding potyviruses. The loci encoding eIF4E are known to be under diversifying selection and each virus evolves so that VPg can bind to eIF4E in the corresponding host [42]. The presence of multiple *eIF4E* alleles generated by diversifying selection may effectively protect plant populations from potyvirus infection, since viruses that have adapted to a host that harbors an *eIF4E* allele often lose infectivity to plants with other alleles. In contrast, no information is currently available regarding how dominant virus resistance genes evolve against viruses. Products of dominant resistance genes interact, whether directly or indirectly, with viral factors (avirulence factors) for resistance. Resistance-breaking virus mutants emerge by mutations that escape the inhibitory interaction with the resistance factor. Even if mutations occur in dominant resistance loci to generate diversified alleles, most of the alleles would not be useful to counter escaped viruses since gaining the ability to interact with new factor is much more difficult than losing an established interaction. Therefore, diversification may not be equally effective for the evolution of dominant resistance genes as for recessive resistance genes.

In this study, we found that a small region (residues 79–112) of the dominant resistance gene *Tm-1* has been under positive selection in *S. habrochaites* (Figure 2). The positively selected region is important to inhibit ToMV RNA replication [15], and an amino acid substitution in this region (I91T) extends the antiviral spectrum (Figure 4). In addition, the amino acid sequences under positive selection were grouped into two groups corresponding to ToMV resistance phenotypes (Figure 3). These observations suggest that infection by tobamoviruses served as a selective pressure during *S. habrochaites* evolution. Although little information regarding ToMV strains infecting wild *S. habrochaites* population is currently available, the results of the experimental evolution analyses suggest that ToMV easily evolves to escape from the inhibition by *Tm-1* alleles. Thus, ToMV and the resistance gene *Tm-1* have likely coevolved.

**Figure 8:**
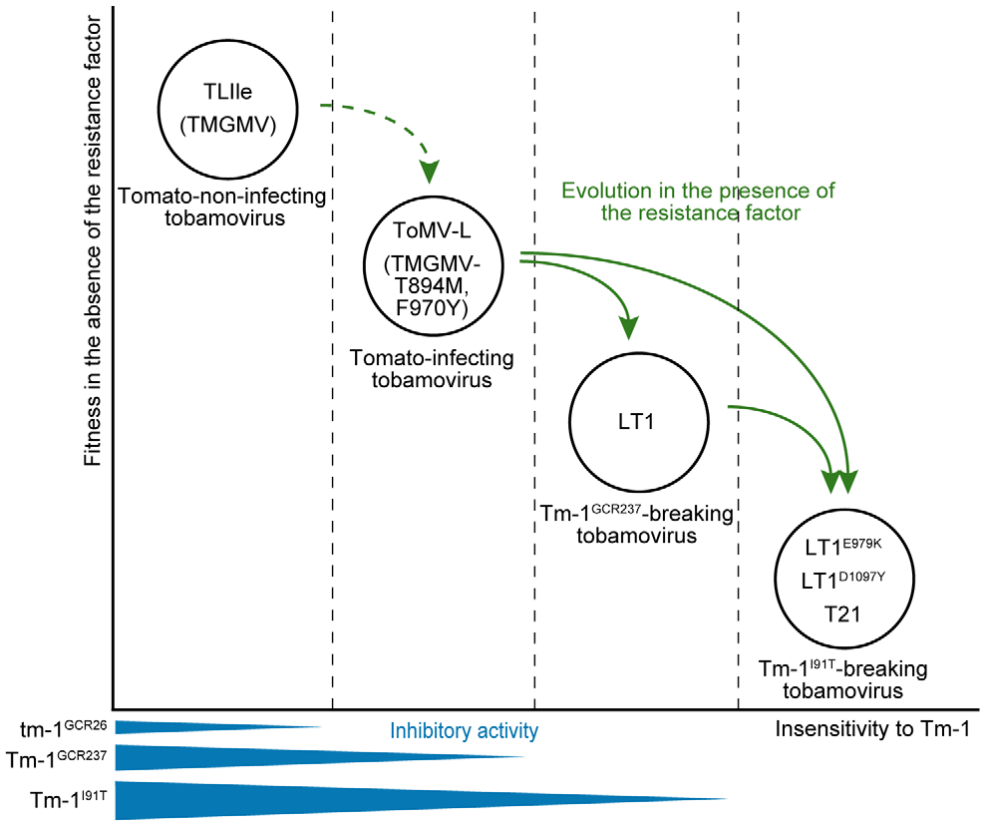
Model of the hierarchical interactions between ToMV and *Tm-1*. The horizontal axis indicates the insensitivity (i.e., weakness of binding to) of the tobamovirus replication proteins to Tm-1. The vertical axis indicates fitness of tobamoviruses in *Tm-1*-lacking hosts. The dashed lines represent thresholds that determine whether viruses can infect plants harboring *tm-1^GCR26^*, *Tm-1^GCR237^*, or *Tm-1^I91T^*. Schematic representation of the inhibitory activities of tm-1^GCR26^, Tm-1^GCR237^, and Tm-1^I91T^ are shown at the bottom. Whether ToMV-L evolved from a tm-1-sensitive prototype remains unknown, although TMGMV evolved to TMGMV-T894M,F970Y in the presence of tm-1^GCR26^ with apparent fitness costs [30], [41].

We demonstrated that interactions between ToMV mutants and Tm-1 variants are hierarchical. The hierarchical classification may also apply to other tobamoviruses; the multiplication of tm-1^GCR26^-sensitive TMGMV and PMMoV are also inhibited by Tm-1^GCR237^ [30], and a TMGMV mutant that can replicate in the presence of tm-1^GCR26^ (TMGMV-T894M,F970Y) cannot overcome the resistance by Tm-1^GCR237^ (K.I. and M.I., unpublished result). Thus, wild-type TMGMV and TMGMV-T894M,F970Y are categorized into the TLIle class and ToMV-L class, respectively (Figure 8). Based on these considerations and the results of *in vitro* RNA replication inhibition by Tm-1 variants, we suggest that the relative strengths of binding to the replication proteins and inhibition of RNA replication by each Tm-1 protein variant decreases in the order of TLIle, TMGMV>ToMV-L, TMGMV-T894M,F970Y>LT1>T21, LT1^E979K^, and LT1^D1097Y^ (Figure 8). Additionally, for each ToMV variant, the binding strengths to the replication proteins and inhibition of RNA replication by Tm-1 variants decrease in the order of Tm-1^I91T^>Tm-1^GCR237^>tm-1^GCR26^ (Figure 8). Under selective pressure by tobamoviruses, *Tm-1* may have modified the strength of its inhibitory activity, but not diversified the recognition spectra.

Currently, two modes of pathogen–host coevolution have been proposed: an ‘arms race’ model in which short-lived alleles are repeatedly fixed in both pathogens and hosts, and a ‘trench warfare’ model in which balanced polymorphisms in relevant genes are maintained [45]–[47]. With regard to the ToMV resistance, the only known function of the *Tm-1* gene to date, the resistant alleles in *S. habrochaites* would be more beneficial than susceptible ones and thus the susceptible alleles could be eliminated in the ‘arms race’ model. However, in this study, we found that 45 of 149 *S. habrochaites* plants from 24 accessions permit efficient ToMV-L multiplication (Table 1), and the positively selected region of 19 of the 48 sequences of *Tm-1* cDNA have identical or very similar sequences to those of ToMV-L-susceptible plants (Figure S1). Although some biases may have resulted from the seed collection and propagation processes, ToMV-susceptible *S. habrochaites* plants should exist to some extent in nature. Considering that the region under positive selection of the putative ToMV-L-susceptible *Tm-1* alleles have very low amino acid sequence diversity (Figures 3 and S1), the alleles may also be adaptive and maintained by balancing selection. Possible driving forces of balancing selection includes costs of resistance; i.e., the ToMV-L susceptible allele may be beneficial in particular situations regarding the original function of *Tm-1* or show resistance to other (tobamo)viruses. On the other hand, for ToMV, overcoming *Tm-1* resistance was associated with fitness costs (Figure 7), which may help avoid fixation of the resistance-breaking mutations in the ToMV population, especially when the viruses frequently infect ToMV-L-susceptible *S. habrochaites* subpopulations. Taken together, ToMV and *Tm-1* may have been under a coevolutionary process following a trench warfare-like model.

The above speculation predicts that *Tm-1*-sensitive (ToMV-L class) and resistance-breaking (LT1 class or higher hierarchy) ToMV strains should coexist in nature. Thus, even if *S. habrochaites* evolves a new resistance allele that inhibits multiplication of resistance-breaking strains, it may not be very beneficial unless it maintains the ability to inhibit lower hierarchy strains. This may explain, at least in part, why *Tm-1* appears to have evolved to strengthen its inhibitory activity but does not produce diversified alleles that have different antiviral spectra. Such an evolutionary process of a resistance gene and subsequent viral escape would result in hierarchical interactions. Similar hierarchical interactions were observed between tobamoviruses and the *L* gene alleles of pepper [18]. The *L* gene recognizes the CP of tobamoviruses and elicits defense reactions [24]–[26]. In addition, a recent report showed that the ability of tobamoviruses to overcome *L* alleles is associated with high fitness costs [48]. Thus, regardless of the mechanisms of action, coevolutionary processes that we proposed above for ToMV and *Tm-1* may often occur between viruses and the corresponding dominant resistance genes.

To conclude, we would like to discuss from a practical view. In the *in vitro* system, increased amounts of Tm-1 protein enhance the inhibition of ToMV RNA replication (Figure 6). In *Tm-1/tm-1* heterozygous plants, resistance-breaking ToMV mutants emerge more frequently than in *Tm-1/Tm-1* homozygous plants [36], indicating that the intracellular level of the Tm-1 protein influences durability. In addition, *Tm-1^I91T^* inhibits ToMV RNA replication more strongly than *Tm-1^GCR237^* (Figure 6). Results of the competition assay suggest that a correlation exists between the level of RNA replication inhibition by Tm-1 and fitness costs (Figure 7), i.e., a higher quality and/or quantity of the Tm-1 protein are associated with increased fitness costs for ToMV to overcome the resistance (Figure 8). Thus, one effective strategy to create durable or sustainable tobamovirus-resistant crops would be to identify stronger *Tm-1* alleles from genetic resources or create such alleles by mutagenesis and subsequent overexpression of these genes.

## Materials and Methods

### Viruses

For inoculation into *S. habrochaites*, crude leaf homogenates (50 mg of leaf tissues in 1 ml of 5 mM sodium phosphate buffer, pH 7) of ToMV-L [49] or LT1 [20] infectious transcript-inoculated tomato were mechanically inoculated onto the first or second true leaves. The infectious cDNA clone of TLIle [50] was provided by Dr. Yuichiro Watanabe (the University of Tokyo), those of LT1^E979K^ and LT1^D1097Y^ were created by site-directed mutagenesis, and that of T21 was created by replacing the region encompassing the mutation sites of pTLW3 [49] with the RT-PCR-amplified fragment from the genome of L_11_A237, a ToMV mutant capable of overcoming *Tm-1* (MAFF260005, obtained from the NIAS Genebank), which has the same mutations as ToMV1-2 [28] (Figure 1). *In vitro* transcripts synthesized from the infectious clones using an AmpliCap T7 High Yield Message Maker kit (CELLSCRIPT, Inc., Madison, WI) were used for electroporation, *in vitro* translation/replication, and co-inoculation onto GCR26 leaves.

### Plants

The seeds of *S. habrochaites* accessions were obtained from GRIN. Plants were grown at 24°C under a 16-h light/8-h dark cycle. Tobacco BY-2 cells were grown, maintained, and transformed as described previously [51], [52]. For transformation of BY-2 cells, tm-1^GCR26^-FLAG, Tm-1^GCR237^-FLAG [15], and Tm-1^I91T^-FLAG (created by site-directed mutagenesis from Tm-1^GCR237^) cDNA were cloned into the binary vector pBI121.

### Analysis of *Tm-1* cDNA of *S. habrochaites*

RT-PCR was performed using RNA extracted from leaves of a randomly chosen individual of each *S. habrochaites* accession as a template with the following primers: 5′-tccattttgaaatctcgattgtaaca-3′ and 5′-taaagaaagaggtgaagaccataca-3′. The amplified fragments were sequenced directly as well as after cloning to obtain two sequences from a diploid individual. Some plants showed no polymorphisms in the coding region, which we assumed to be homozygous. Accession numbers of the sequences that were deposited in the DDBJ/EMBL/GenBank nucleotide sequence databases are AB713134–AB713181. Obtained sequences were analyzed by PERMUTE in the OMEGAMAP package [33] to examine a correlation between distance and linkage disequilibrium, and by OMEGAMAP [33] to detect natural selection in the presence of recombination. OMEGAMAP analysis was conducted using 10 randomly chosen orderings of the haplotypes and the following priors: μ = Improper inverse, κ = Improper inverse, φ = Improper inverse. For ω, we used inverse distribution with a range of 0.001–100 and set the average length of blocks for ω at 30 codons. For ρ, we used inverse distribution with a range of 0.001–100 and set the average length of blocks for ρ at 80 codons. The inverse distribution corresponds to a uniform distribution on the log scale. We assumed that all codons have equal frequencies. Two independent Markov chain Monte Carlo chains were run for 500,000 iterations, with a 25,000 iteration burn-in. Upon convergence the two chains were merged to infer ω. Tajima's *D* was calculated by DNAsp ver. 5.1 [53] using 120-bp window slides in steps of 30 bp. Although seed propagation processes of each accession would reduce the genetic diversity of the population and could affect the analyses, we considered this effect to be negligible since several alleles from different accessions have identical or very similar sequences, and we sequenced the *Tm-1* cDNA of only one individual from each accession. The positively selected region in the *Tm-1* cDNA of five *S. habrochaites* plants that did not accumulate ToMV-L CP (i.e., resistant plants) and five plants that accumulate ToMV-L CP (i.e., susceptible plants) were sequenced as described above. Each of these plants had a single sequence in this region as shown in Figure 3.

### Protoplast experiments

Isolation of protoplasts from tobacco BY-2 cells followed by electroporation of viral RNA and preparation of mdBYL was performed essentially as described previously [38], [51], [54]. For detection of CP, approximately 5×10^5^ protoplasts were inoculated with 2 µg of ToMV genomic RNA. CP accumulation at 20 hpi was examined by SDS-PAGE followed by Coomassie blue staining. For the competition assay, approximately 5×10^5^ protoplasts were inoculated with mixtures of genomic RNA (3 µg each) from two ToMV variants or 6 µg of a single variant. The protoplasts inoculated with a single variant were mixed with those with another variant and cocultured for 20 hours. RNA was extracted from one-tenth of the inoculated protoplasts and the cDNA fragments encompassing the mutation sites were amplified by RT-PCR. Sequencing of the amplified cDNA fragment using GS-FLX titanium was performed by Takara Bio Inc. (Shiga, Japan).

### Immunoprecipitation

Immunoprecipitation analysis of FLAG-tagged Tm-1 variants was performed essentially as described previously [30]. The protein samples were analyzed by Western blotting using anti-ToMV replication protein antibody [52] and anti-FLAG antibody (Sigma, St. Louis, MO).

### *In vitro* translation and replication reactions of viral RNA

Messenger RNAs for tm-1^GCR26^, Tm-1^GCR237^, or Tm-1^I91T^ were synthesized from the plasmids harboring the corresponding cDNAs [15] using the mScript mRNA Production System (CELLSCRIPT, Inc.). The messenger RNAs (64 fmol/µl of reaction mixture) or tobamovirus RNA (7.12 fmol/µl of reaction mixture) were translated in mdBYL-based translation mixtures [38], [51] at 23°C for 1 hour. Tobamovirus RNA-translated mixtures (1 µl) were mixed with a mock-translated mixture (14 µl), translation mixture for Tm-1 variants (1 µl) plus mock-translated mixture (13 µl), translation mixture for Tm-1 variants (4.67 µl) plus mock-translated mixture (9.33 µl), or 14 µl of translation mixture for Tm-1 variants, and incubated at 23°C for 20 minutes. The mixtures were further incubated with 5 µl of P30 BYL (membrane fraction of BYL) at 15°C for 2 hours, followed by incubation with 5 µl of ribonucleoside triphosphate mixture containing [α-^32^P]CTP [51] at 23°C for 1 hour. The reaction was terminated by phenol extraction, and the RNA products were purified and analyzed by electrophoresis in an 8 M urea–2.4% polyacrylamide gel and autoradiography.

## Supporting Information

Figure S1 **Amino acid sequences under positive selection in the Tm-1 protein of** ***S. habrochaites*** **.** 48 amino acid sequences of the Tm-1 protein from 24 *S. habrochaites* accessions were aligned. The positively selected region (79–112) is indicated. Identical amino acid residues to those of Tm-1^GCR237^ are indicated by dots. a and b indicate two sequences obtained from a single plant. The same sequence is represented twice as both a and b when the plant had no sequence heterogeneity in the indicated region.
(TIF)

Figure S2 **Amino acid sequence alignments of the Tm-1 protein from LT1-resistant** ***S. habrochaites*** **.** Deduced amino acid sequences of the Tm-1 protein from three *S. habrochaites* plant individuals showing the LT1-resistant phenotypes (PI251304, PI365904, PI365906), GCR237 (LT1-susceptible but ToMV-L-resistant), and GCR26 (susceptible to both ToMV-L and LT1) are compared. Common changes in LT1-resistant *S. habrochaites* are highlighted.
(TIF)

Figure S3 **Estimation of relative fitness of a virus variant to the other co-inoculated virus variant in plants.** A model developed for estimation of relative fitness of a virus variant to a co-inoculated virus is schematically shown. The ratios of exclusive infections by one of the two variants and coinfection by the two variants were calculated by this model using different parameter sets, and were compared with the frequencies of exclusive infections and coinfections that were experimentally observed to estimate most likely parameter values for *r*, *λ*_1_, *λ*_2_, and *λ*_3_. The most-likely estimates and their standard errors or standard deviations are also shown in a table. See Text S1 for detailed procedures.
(TIF)

Table S1 **Pyrosequencing examinations of the proportion of viral strains accumulated in co-inoculated protoplasts.**
(DOCX)

Text S1 **Estimation of relative fitness of ToMV derivatives in co-inoculated tomato plants.**
(DOCX)
