# Supplementary figures and images for: Coevolution and Hierarchical Interactions of Tomato mosaic virus and the Resistance Gene Tm-1
Source: PLoS Pathog. 2012 Oct 18;8(10):e1002975. doi: 10.1371/journal.ppat.1002975 (PMC3475678; doi:10.1371/journal.ppat.1002975)

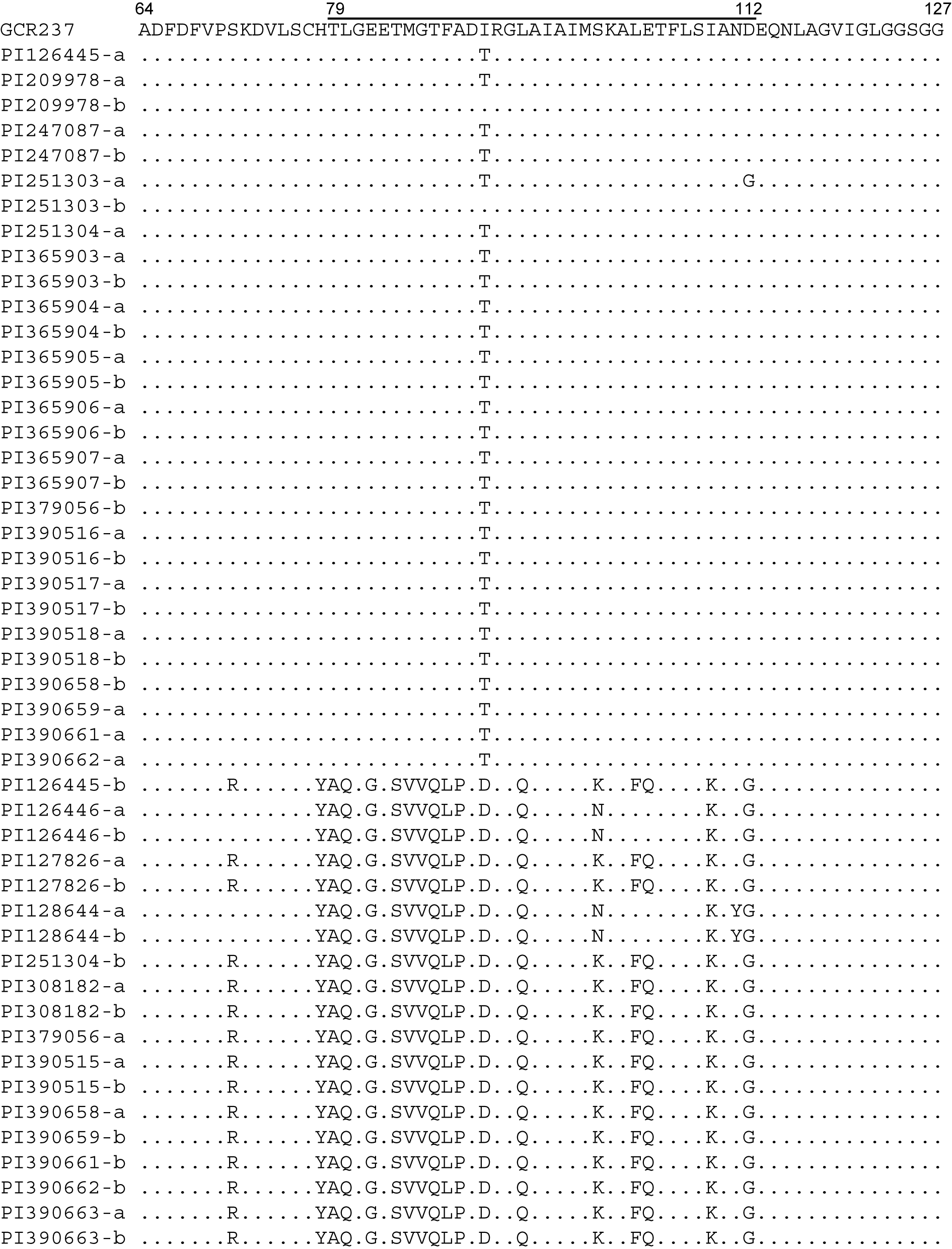

Supplement: Figure S1 — Amino acid sequences under positive selection in the Tm-1 protein of S. habrochaites . 48 amino acid sequences of the Tm-1 protein from 24 S. habrochaites accessions were aligned. The positively selected region (79–112) is indicated. Identical amino acid residues to those of Tm-1GCR237 are indicated by dots. a and b indicate two sequences obtained from a single plant. The same sequence is represented twice as both a and b when the plant had no sequence heterogeneity in the indicated region. (TIF) [file ppat.1002975.s001.tif]

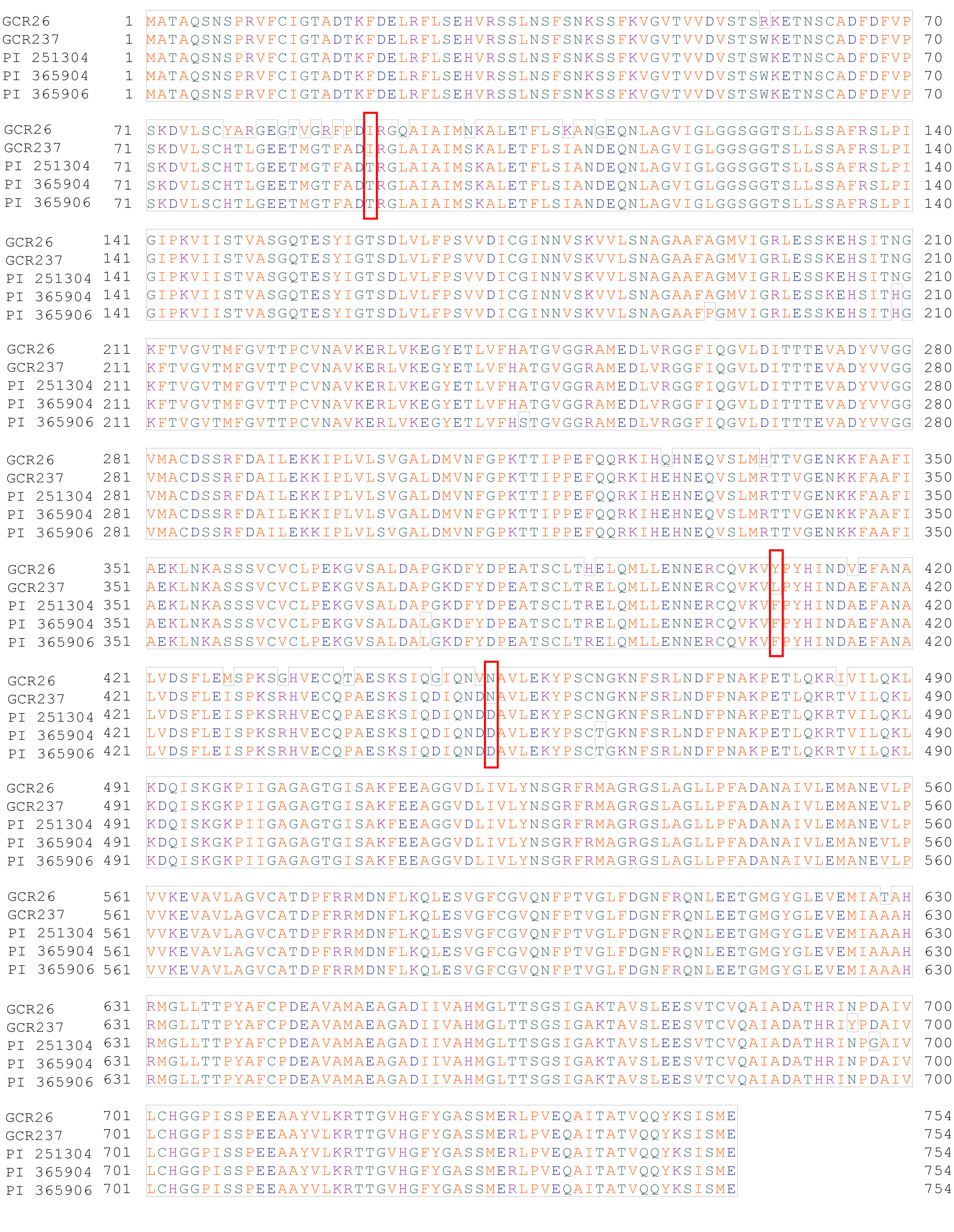

Supplement: Figure S2 — Amino acid sequence alignments of the Tm-1 protein from LT1-resistant S. habrochaites . Deduced amino acid sequences of the Tm-1 protein from three S. habrochaites plant individuals showing the LT1-resistant phenotypes (PI251304, PI365904, PI365906), GCR237 (LT1-susceptible but ToMV-L-resistant), and GCR26 (susceptible to both ToMV-L and LT1) are compared. Common changes in LT1-resistant S. habrochaites are highlighted. (TIF) [file ppat.1002975.s002.tif]

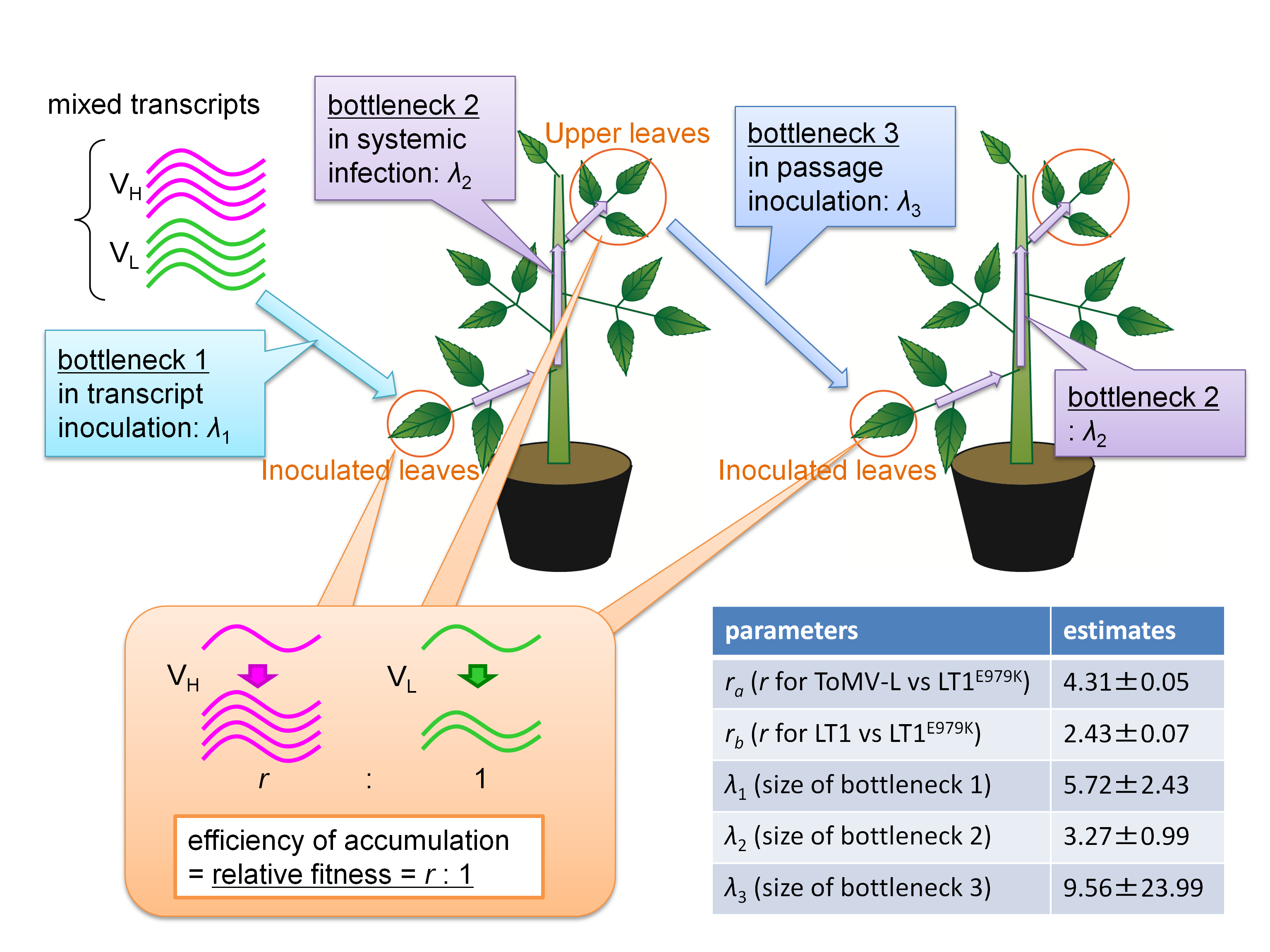

Supplement: Figure S3 — Estimation of relative fitness of a virus variant to the other co-inoculated virus variant in plants. A model developed for estimation of relative fitness of a virus variant to a co-inoculated virus is schematically shown. The ratios of exclusive infections by one of the two variants and coinfection by the two variants were calculated by this model using different parameter sets, and were compared with the frequencies of exclusive infections and coinfections that were experimentally observed to estimate most likely parameter values for r, λ 1, λ 2, and λ 3. The most-likely estimates and their standard errors or standard deviations are also shown in a table. See Text S1 for detailed procedures. (TIF) [file ppat.1002975.s003.tif]
