# Supplementary material for: Coevolution and Hierarchical Interactions of Tomato mosaic virus and the Resistance Gene Tm-1
Source: PLoS Pathog. 2012 Oct 18;8(10):e1002975. doi: 10.1371/journal.ppat.1002975 (PMC3475678; doi:10.1371/journal.ppat.1002975)
Supplement: Table S1 — Pyrosequencing examinations of the proportion of viral strains accumulated in co-inoculated protoplasts. (DOCX) [file ppat.1002975.s004.docx]

**Table S1. Pyrosequencing examinations of the proportion of viral strains accumulated in co-inoculated protoplasts.**

| **Virus A** |  | **Virus B** | | | | |
| --- | --- | --- | --- | --- | --- | --- |
|  |  | **ToMV-L** | **LT1** | **T21** | **LT1^E979K^** | **LT1^D1097Y^** |
| **TLIle** | Coinfection | [351:351] | [350:304] | [376:220] | [356:270] | [396:157] |
|  | Control | (694:800) | (616:836) | (730:647) | (715:618) | (651:600) |
|  | Ratio | 1:0.87 | 1:0.64** | 1:0.66** | 1:0.88 | 1:0.43** |
| **ToMV-L** | Coinfection |  | [305:310] | [122:25] | [344:231] | [508:176] |
|  | Control |  | (618:765) | (398:254) | (719:465) | (806:672) |
|  | Ratio |  | 1:0.82* | 1:0.32** | 1:1.04 | 1:0.42** |
| **LT1** | Coinfection |  |  | [310:171] | [387:252] | [170:66] |
|  | Control |  |  | (561:508) | (783:543) | (428:367) |
|  | Ratio |  |  | 1:0.61** | 1:0.94 | 1:0.45** |
| **T21** | Coinfection |  |  |  | [308:342] | [408:271] |
|  | Control |  |  |  | (847:705) | (747:674) |
|  | Ratio |  |  |  | 1:1.33** | 1:0.74** |
| **LT1^E979K^** | Coinfection |  |  |  |  | [462:185] |
|  | Control |  |  |  |  | (733:828) |
|  | Ratio |  |  |  |  | 1:0.35** |

Coinfection: counts for each virus [virus A: virus B] in the progeny of coinfected non-transgenic BY2 protoplasts by viruses A and B. Control: counts for the progeny of individually infected and cocultured protoplasts (virus A: virus B). Ratio: normalized ratio (ratio of coinfection/ ratio of individual infection). Values for virus A = 1. *: *p*<0.05, **: *p*<0.01 based on a chi-square test for the ratio of the two strains in coinfection against the ratio expected from the individual infection.
