# Supplementary material for: Coevolution and Hierarchical Interactions of Tomato mosaic virus and the Resistance Gene Tm-1
Source: PLoS Pathog. 2012 Oct 18;8(10):e1002975. doi: 10.1371/journal.ppat.1002975 (PMC3475678; doi:10.1371/journal.ppat.1002975)
Supplement: Text S1 — Estimation of relative fitness of ToMV derivatives in co-inoculated tomato plants. (DOCX) [file ppat.1002975.s005.docx]

**Text S1**

**Estimation of relative fitness of ToMV derivatives in co-inoculated tomato plants**

To estimate the relative fitness between two co-inoculated variants in the two sets of experiments (ToMV-L vs. LT1^E979K^ and LT1 vs. LT1^E979K^) (Figure 7B), we developed a simple model as follows (Figure S3): We assumed that, in all the leaflets co-infected with the two variants, the variant with higher fitness (V_H_) accumulates with *r*-fold efficiency to the variant with lower fitness (V_L_) (r_a_ for ToMV-L vs. LT1^E979K^; r_b_ for LT1 vs. LT1^E979K^; *r_a_* ≥ 1; *r_b_* ≥ 1); we defined *r_a_* and *r_b_* as relative fitness for the respective sets of experiments. In an initially inoculated leaflet, biased accumulation modeled above occurs, and after that, viruses move systemically to upper leaves and establish infection. Viruses in upper leaflets again accumulate with biased efficiencies, which were then used for passage inoculation. In the actual experiments, we used four or more leaflets for passage inoculation; however, we here assumed that one leaflet was used for inoculation, because this assumption can be considered to have limited effect on the estimation of relative fitness (see below). In the passage-inoculated leaflets, viruses again accumulate with biased efficiencies, and move systemically to upper leaves and establish infection. As genetic bottlenecks (i.e., limitations in the number of viral genomes that establish infection) are known to occur in mechanical inoculations and systemic infections of plant RNA viruses [1-3], we assumed bottlenecks for three types of events, 1) initial local infection to a leaflet by mechanical inoculation, 2) systemic infection of a leaflet in upper leaves, and 3) passage inoculation of a leaflet of a new plant; the bottleneck sizes (i.e., the number of genomes that establish infections) were assumed to follow Poisson distribution with the mean *λ*_1_, *λ*_2_, and *λ*_3_, respectively. These genetic bottlenecks isolate variants in a stochastic manner, causing stochastic occurrence of exclusive infections of the leaflets by one of the two variants, which were observed in the experiments (Figure 7B).

By comparing the expected ratio by the model with the experimentally observed frequencies of exclusive infection by V_H_, mixed infection by V_H_ and V_L_, and exclusive infection by V_L_, the most likely values for r_a_, r_b_, *λ*_1_, *λ*_2_, and *λ*_3_ can be simultaneously estimated using the maximum-likelihood method. Calculation was done using statistical computing software R [4], and we obtained the relative fitness *r_a_* = 4.31 ± 0.05 and *r_b_* = 2.43 ± 0.07 (maximum-likelihood estimates ± standard deviations); the mean bottleneck sizes *λ*_1_ = 5.72 ± 2.43, *λ*_2_ = 3.27 ± 0.99, and *λ*_3_ = 9.56 ± 23.99 (maximum-likelihood estimates ± standard errors). Assuming normal distribution of the estimates for *r_a_* and *r_b_*, we can conclude that *r_a_* > *r_b_* > 1, indicating that fitness of ToMV-L or LT1 is higher than LT1^E979K^ and relative fitness of ToMV-L to LT1^E979K^ is higher than that of LT1 to LT1^E979K^. This is consistent with the protoplast experiment in which ToMV-L was shown to have higher fitness than LT1 (Figure 7A). Our estimate for bottleneck size in systemic infection (*λ*_2_ = 3.27 ± 0.99) is also consistent with the previous study about genetic bottleneck in systemic infection of tobacco by *Tobacco mosaic virus*, in which bottleneck size was estimated to be in the order of units [2], suggesting that our simultaneous estimation of the relative fitness and the bottleneck sizes worked properly. The especially large standard error for *λ*_3_ is due to the difficulty in bottleneck size estimation for passage inoculation in the ToMV-L vs. LT1^E979K^ experiments. Because the number of observed frequencies of coinfection had been small before passage inoculation, the frequencies did not decrease after passage inoculation, which leads to large and instable estimation of the bottleneck size. In contrast, the bottleneck size for passage inoculation in the LT1 vs. LT1^E979K^ experiments was calculated as a small number. As a result, likely values for *λ*_3_ took divergent values.

As mentioned above, the number of leaflets used for passage inoculation in the model was considered to have limited effect on the estimation of relative fitness, because, even if we assumed to use an infinite number of leaflets, we obtained *r_a_* = 5.43 ± 0.04 and *r_b_* = 2.97 ± 0.07, which do not change our conclusion; this assumption (i.e., use of an infinite number of leaflets) affected the estimate for bottleneck size in passage inoculation *λ*_3_ = 1.87 ± 0.97, but did not largely affect the other estimates for bottleneck sizes *λ*_1_ = 4.21 ± 1.19 and *λ*_2_ = 3.69 ± 1.27.

**References**

[1] Hall JS, French R, Hein GL, Morris TJ, Stenger DC (2001) Three distinct mechanisms facilitate genetic isolation of sympatric wheat streak mosaic virus lineages. Virology 282: 230-236

[2] Sacristán S, Malpica JM, Fraile A, García-Arenal F (2003) Estimation of population bottlenecks during systemic movement of tobacco mosaic virus in tobacco plants. J Virol 77: 9906-9911

[3] Li H, Roossinck MJ (2004) Genetic bottlenecks reduce population variation in an experimental RNA virus population. J Virol 78: 10582-10587.

[4] R **Development Core Team** (2009) R: a language and environment for statistical computing. R Foundation for Statistical Computing, Vienna, Austria. <http://www.R-project.org>.
